# Impact of Measles, Mumps, and Rubella Vaccination on Hospitalizations and Human Capital: Evidence from Copenhagen School Health Records

**DOI:** 10.3390/vaccines13030302

**Published:** 2025-03-11

**Authors:** Onur Altindag, Jane Greve, Erdal Tekin

**Affiliations:** 1 Department of Economics, Bentley University, Waltham, MA 02452, USA; oaltindag@bentley.edu; 2 Institute of Labor Economics (IZA) and National Bureau of Economic Research (NBER), 53113 Bonn, Germany; jagr@vive.dk; 3 VIVE—The Danish Center for Social Science Research, 1052 Copenhagen, Denmark; 4 School of Public Affairs, American University, Washington, DC 20016, USA

**Keywords:** measles, mumps, and rubella, MMR, vaccine, Denmark, school, vaccination, healthcare, education, economic outcomes

## Abstract

**Background:** This paper investigates the long-term effects of the measles, mumps, and rubella (MMR) vaccine on healthcare, education, and economic outcomes using a novel dataset from the Copenhagen School Health Records Register. **Methods:** To address potential endogeneity, we use within-sibling variation in vaccination status induced by different periods of vaccine availability in Denmark. **Results:** Our findings reveal that, prior to the establishment of herd immunity, vaccinated cohorts experienced substantial protection against hospitalizations related to MMR. During the same period, we also observe modest improvements in educational outcomes and positive—though statistically insignificant—estimates for labor market outcomes among siblings with discordant vaccination status. We find no impact of vaccination on hospitalizations, education, or economic outcomes for cohorts born after herd immunity was established, a period when everyone benefited from herd protection regardless of individual vaccination status. **Conclusions:** MMR vaccination, before herd immunity, reduced hospitalization due to Measles, Mumps, and Rubella. The impact on later outcomes, such as education, income, and employment lacks statistical precision at conventional levels. Evidence suggest positive self-selection in vaccination among families with high socioeconomic status.

## 1. Introduction

The development of vaccines marks one of the most remarkable public health breakthroughs in human history, transforming global health by substantially reducing disease burdens through direct protection against infections and the establishment of herd immunity. A notable example of this achievement is the introduction of the measles vaccine in 1963 in the United States, followed by a combined measles, mumps, and rubella (MMR) vaccine in 1971. Before these vaccines, nearly everyone was infected with major outbreaks every two to three years, with approximately 30 million cases and 2.6 million annual deaths worldwide [1,2] (in the United States alone, it has been estimated that 3 to 4 million people were infected each year, leading to approximately 400 to 500 deaths, 48,000 hospitalizations, and 1000 cases of encephalitis [3]). The introduction of measles vaccine produced immediate and significant benefits, drastically reducing mortality and morbidity associated with the disease. For instance, by 1965, the number of measles cases in the U.S. began to plummet, with only about 22,000 cases reported in 1968—a drop of over 97 percent in just three years. Globally, the World Health Organization estimates that vaccination efforts have averted approximately 57 million deaths globally between 2000 and 2022 [4].

There is an extensive literature in economics suggesting that interventions that improve health early in life might contribute to improvements in human capital later [5,6,7,8]. However, it is proposed that the developmental advantages linked to vaccine-induced health improvements could be particularly strong with the measles vaccine. This suggestion stems from the widespread epidemiological evidence documenting reductions in infectious disease mortality in many parts of the world following measles vaccination, which cannot be solely attributed to preventing acute measles deaths [9,10,11,12]. One explanation for the stronger than expected benefits of the measles vaccine is the concept of “trained immunity”, where vaccines, particularly live-attenuated ones like the measles vaccine, bolster the innate immune system’s response to various pathogens through epigenetic and metabolic changes [13,14,15]. Alternatively, a series of studies hypothesize that it is the concept of “measles-induced immune amnesia”, that leads to the loss of immune memory cells, resulting in excess non-measles morbidity and mortality, which may last up to five years after measles infection [11,16,17]. These discoveries imply that the total benefits of the measles vaccine, as inferred from the wider literature on vaccine impact, may be underestimated.

Recently, these findings have sparked interest in the social sciences literature aimed at understanding the impact of the measles vaccine on human capital and labor market outcomes. Specifically, studies have examined the impact of introducing the measles vaccine or implementing mass measles vaccination campaigns in several countries, including the United States [18,19], the United Kingdom [20], Mexico [21], and South Africa [22] (the vast majority of the literature on the effects of childhood vaccination focuses on mortality rather than other outcomes—we do not discuss this literature in the interest of space). Atwood [18] shows that the introduction of the measles vaccine in the United States caused increases in earnings and employment among cohorts after the licensing of the vaccine, where the increase in earnings was driven by greater productivity rather than an increase in hours worked. Barteska et al. [19] also explores the United States context, emphasizing the consequences of the mass vaccination campaign introduced four years after the licensing of the measles vaccine. Their paper considers educational attainment rather than solely focusing on labor market outcomes. Their results suggest that the mass vaccination campaign led to an increase of 0.1 years in education among the affected cohorts, albeit only among males. Atwood and Pearlman [21] investigated the impact of a nationwide measles vaccination program in Mexico on the human capital and labor market outcomes of men. They documented substantial increases in educational attainment, employment, and income due to the program with effect sizes two to ten times greater than those found in the U.S. In the context of the United Kingdom, van den Berg et al. [20] showed that exposure to vaccination in early childhood has no influence on years of education. They found a positive effect on adult height, but only among those possessing higher levels of genetic endowment for height, as identified by polygenic scores. Finally, Anekwe et al. [22] examined the impact of measles vaccination on education levels among children in a poor and rural South African community. We identified several studies examining the relationship between vaccination and cognitive and human capital outcomes in developing countries—using propensity score matching, Nandi et al. [23] found that children vaccinated against measles have improved child anthropometry, cognition, and schooling outcomes in Ethiopia, India, and Vietnam. Bloom et al. [24] showed that full vaccination against measles, polio, and tuberculosis is associated with improvements in cognitive test scores in the Philippines, also using propensity matching to control for selection into vaccination status. Lastly, Driessen et al. [25] examined the phased implementation of a vaccination campaign in Bangladesh and showed that vaccination led to increased school enrolment among boys. However, these studies may not fully account for the potential endogeneity stemming from measles exposure or the vaccination decision). This study differs from the others in that it employs a mother fixed-effects model to account for the characteristics that may influence the vaccination decision of parents such as risk attitudes and future aspirations. Using variation based on a sample of 607 children who shared the same mother, but differed in measles vaccination status, the study revealed that measles vaccination increases the average school grade attainment by 0.188 grades.

In this study, we examined a range of human capital and healthcare outcomes to evaluate the long-term impacts of MMR vaccination. Human capital is captured by various measures of education and labor market productivity derived from Danish administrative registries spanning 1977 to 2018. Specifically, educational measures include an indicator for not completing ten years of mandatory education by age 16, an indicator representing those whose highest level of education is ten years or less by age 21, and an indicator for being not in education, employment, or training (NEET) at age 21. Labor market outcomes include an indicator for employment status, annual labor market income, and an indicator for the receipt of social benefits, all measured at age 24. These outcomes collectively capture important aspects of human capital, reflecting both educational attainment and long-term economic engagement [26,27,28,29]. For healthcare, we assess hospitalization due to MMR by age 5 and between ages 2 and 12, obtained from administrative records from 1977 to 2006. These data are combined with a unique dataset from the Copenhagen School Health Records Register to evaluate the impact of MMR vaccination on health outcomes for birth cohorts born between 1977 and 1986. Employing a sibling fixed-effects approach, our research design relies on within-family variation in vaccination status driven by differences in vaccine availability over time. By focusing on periods before and after the establishment of herd immunity in Denmark, this design mitigates the selection bias and enables the causal estimation of the effects of MMR vaccination on hospitalizations, as well as educational and labor market outcomes. Overall, this study provides new insights into both the direct and indirect impacts of MMR vaccination. By examining cohorts exposed to varying levels of herd immunity, we contribute to understanding the broader implications of vaccination, including its influence on health, educational, and economic outcomes.

## 2. Materials and Methods

### 2.1. The Introduction of the Measles Vaccine in Denmark

The combined version of the measles, mumps, and rubella (MMR) vaccine was incorporated into the Danish Vaccination Program (DCVP) on 1 January 1987. Before 1987, 98 percent of the population had contracted measles in Denmark, but this proportion significantly decreased following the introduction of the MMR vaccine [30].

Denmark introduced the MMR vaccine relatively late compared to neighboring countries such as Sweden, Finland, and Norway, where it had been in use since the early 1980s [31]. The delayed adoption of the MMR vaccine in Denmark stemmed from various factors, including the perception of measles, mumps, and rubella as less urgent health threats compared to other diseases. Additionally, Denmark’s reliance on rubella screening programs rather than vaccinations created a path dependency that hindered the integration of new vaccine-based strategies. Institutional inertia, along with political and economic factors further contributed to the delay. However, changes in international health policy during the 1980s, focusing on cost-effectiveness and broader public health benefits, eventually persuaded Danish politicians to include the MMR vaccine in the DCVP.

Prior to the introduction of the MMR vaccine, vaccines against diseases like diphtheria, tetanus, pertussis, and polio were already included in the program. The MMR vaccine had been available for several years before it was officially introduced into the national program, with parents often paying for it out of pocket. The measles vaccine was additionally recommended for high-risk children (EPI-NEWS, 11/1982), leading to ambiguity in the expected direction of self-selection. On one hand, the cost of the vaccine (as it was not free) likely encouraged positive self-selection, whereby healthier children from families with higher socioeconomic status were more likely to be vaccinated. On the other hand, the recommendation for high-risk groups may have resulted in children with poorer baseline health being more frequently vaccinated. Official statistics, which are consistent with our analysis, suggest that the effect of positive self-selection was more pronounced. For instance, in 1983, the Statens Serum Institute distributed 9416 doses of the measles vaccine, 3734 doses of the mumps vaccine, and 653 doses of the rubella vaccine. In the years leading up to 1987 between 15,000 and 25,000 children were vaccinated (EPI-NEWS, 48/1986). The majority of these doses were administered to healthy children at the request of their parents, rather than to those in high-risk groups [32].

Vaccines within the DCVP are provided free of charge and administered by general practitioners (GPs). According to the Danish Health Care Act (Sundhedsloven), all children residing in Denmark are offered seven preventive GP visits (known as Børneundersøgelser) before beginning school. These visits are scheduled at five weeks, five months, one year, two years, three years, four years, and five years of age. During the initial visit after birth, the GP informs parents about the immunizations included in the program. Since the first MMR vaccination is given at 15 months of age, parents must arrange an additional appointment with their primary care physician, beyond the seven preventive visits. Prior to 1987, parents were required to pay for the MMR vaccination, with the cost of one dose of MMR vaccine in 1985 being DKK 110 (including tax), equivalent to around DKK 225/USD 31 in 2020, excluding any GP fees (EPI-NEWS, 44/1985). The fees charged by GPs for administering vaccinations varied ranking from DKK 150 to 300 in 2020. Adjusting this cost to 1985 prices, the fee accounts for approximately half of the vaccine’s price.

Upon the introduction of the MMR vaccination into the DCVP, there was a short transition period lasting approximately one year. During this phase, all children aged 2–12 who had not previously contracted measles or mumps were offered the vaccine, leading to a large-scale immunization effort [33]. From 1987 to 2016, the National Board of Health advised MMR immunizations at 15 months (MMR1) and 12 years (MMR2) (A booster dose (MMR2), administered at the age of 4, was introduced in 2008. The rationale for the change was to increase herd immunity by increasing the immunity of children aged under 12 years [34]).

### 2.2. Data

#### 2.2.1. Copenhagen School Health Records Register

The Copenhagen School Health Records Register (CSHRR) contains comprehensive health data collected through school health examinations [35]. The data in the CSHRR were primarily collected during mandatory school health examinations conducted at the entry and exit points of school education in Copenhagen (the school health records only exist for Copenhagen—approximately 20% of the Danish population lives in Copenhagen). The Danish National Health Service Registry (DNHSR) started recording MMR vaccine data from 1990 onward. However, prior to 1996, procedural constraints prevented the direct linkage of services to children under 16 years old. Consequently, detailed information on the immunization status of individuals born before, during, and after the introduction of the MMR vaccine in Denmark in 1987 was previously unavailable. To improve data accessibility, we undertook a substantial effort to digitize individual vaccination records from the physical files of the Copenhagen School Health Record Register for cohorts born between 1977 and 1994 [32]. This process involved a detailed history taken from parents regarding their child’s health and vaccination record. We digitized the physical files of these records, including detailed vaccine information, enhancing the accessibility and usability of the data for research purposes. As a result of these efforts, the CSHRR now includes data on the type of MMR vaccination administered (MMR1 or MMR2), along with the dates of these vaccinations. The data were described in detail, compared with vaccine information available from reimbursement records for later cohorts, and validated in Altindag et al. [32].

We use the vaccine information for cohorts from 1977 to 1994. The raw data include 68,239 children. Following the approach in Altindag et al. [32], we exclude children from our study who had missing information about all vaccines, as can be seen in Altindag et al. [32]. The children with missing information on any additional vaccines other than MMR mainly consist of children whose parents did not provide any vaccine information in their children’s health card. Removing the children with no information on any vaccines does not introduce significant selection into the sample. This resulted in a total of 58,735 observations remaining in our sample. Furthermore, as a sizeable proportion of immigrants not born in Denmark have missing information on immunizations, we exclude these observations, focusing on 52,816 children born in Denmark in our main analysis. Although excluding children not born in Denmark makes the sample more comparable to the full Danish population, we acknowledge that it is not fully representative of the entire population in Denmark.

As we describe in the empirical strategy, our primary research design relies on within-sibling comparisons in the MMR vaccination status to mitigate the bias arising from the potential endogeneity of vaccination decisions. In our full sample of 52,816 children, 27,734 are siblings born to 12,452 unique mothers. Among sibling pairs born before 1987, 30% have discordant vaccination statuses. For sibling pairs that include siblings born both before and after 1987, 24% have discordant vaccination statuses. In sibling pairs born after 1987, 17% display differing vaccination statuses (see Table A1).

#### 2.2.2. Healthcare, Education, and Labor Market Outcomes

We link our samples from the CSHRR to several administrative registers maintained by Statistics Denmark using a unique civil registration number, which facilitates connections between children and their parents and siblings. For the children, we include information on birth year, gender, birth order, and parental education, with parental information measured in the year before birth.

Using extensive records on hospital admissions and diagnoses from 1977 through 2006, we construct binary indicator variables for being hospitalized with MMR before age 5 and at ages between 2 and 12, respectively. The later age-restriction is motivated by the age-range where the children receive the MMR vaccination (age 15 months and 12 years).

Next, we construct the measures of educational and labor market outcomes for the children using administrative registries from 1977 through 2018. The educational outcomes include an indicator for not completing ten years of education with a final exam before age 16. In Denmark, it is mandatory to receive at least 10 years of education; however, approximately 10% do not complete the final exam after 10 years of education [36]. This indicator is a measure of the expected long run human capital outcome as these children have a high probability of not completing any further education [36]. As individuals with no formal education beyond basic education tend to perform less successfully in the labor market, the lack of formal education is widely regarded as a significant economic and social issue in most countries. Therefore, we constructed an indicator representing those whose highest completed education is ten years or less by the age of 21. Similarly, we created an indicator for whether individuals are employed or pursuing further education, meaning that they are not part of the group categorized as not in education, employment, or training (NEET) at the age of 21.

We consider three labor market outcomes measured at age 24. These include an indicator for being employed (where the main income during the year is from wage or self-employment), annual gross wage income (we use the inflation-adjusted annual gross wage, with the baseline year set as 2018), and an indicator for receiving social benefits, such as unemployment or social security benefits. While the first two outcomes serve as indicators of long-term economic well-being, we are limited by measuring these outcomes at the age of 24, the age at which labor market data are available for all cohorts (we have access to registry data up until 2018). At this age, some individuals may still be enrolled in education and therefore not yet employed or earning an income. The latter outcome, which measures the probability of receiving social benefits, reflects a relatively weak attachment to the labor market and offers an additional way to assess long-term economic consequences.

Table 1 presents the descriptive statistics for the child and mother characteristics and the outcome variables for both the full sample and the sibling sample included in the analysis. Both samples exhibit similar child characteristics and socioeconomic outcomes: 49% of the children are female, both have an identical dropout rate before grade 9 (12%), and the same proportion of youths active at the age of 21 (83%). The key differences lie in family structure due to the sibling sample only consisting of families with multiple children. The sibling sample has more children per family (2.36 vs. 1.71), and mothers who are more likely to be married (63% vs. 58%). While variables related to family composition and maternal characteristics show statistically significant differences, the two samples are largely comparable in terms of children’s hospitalization, education, and labor market outcomes.

### 2.3. Empirical Strategy

We employ a within-sibling difference-in-differences approach to estimate the short- and long-run effects of MMR immunization among children in our sample. The distinct phases of vaccination in Denmark allow for a unique comparison of siblings with discordant vaccination statuses across two periods: 1977–1986 and 1987–1994.

The first period includes cohorts born in 1977 through 1986, during which the MMR vaccine was not part of the national immunization program. Although the vaccine was available on the market, uptake was low, and herd immunity had not been established. Consequently, all children born during this period were exposed to circulating measles, mumps, and rubella viruses, with only vaccinated children receiving individual protection.

The second period covers birth cohorts from 1987 to 1994. During this time, a large-scale immunization campaign was launched, and the MMR vaccine became freely available through the national immunization program. Due to high vaccination rates and the establishment of herd immunity, most children born after 1986 were not exposed to measles, mumps, or rubella viruses, regardless of their individual vaccination status. Therefore, only cohorts born between 1976 and 1986 are expected to exhibit any effects from the MMR vaccine, as they were exposed to circulating pathogens. Therefore, the post-1986 herd immunity period offered an opportunity to perform a placebo test, where any observed effects, including the primary health impacts of the MMR vaccine, should diminish or disappear.

Moreover, our approach relies on sibling fixed effects, which substantially mitigates any bias from self-selection into vaccine uptake. Since, most often, the same parents make the immunization decision for all siblings, the decision should primarily be influenced by time-varying conditions, likely due to supply-side factors, rather than by socio-economic and cultural characteristics that might be correlated with both vaccination status and later-life health and economic outcomes.

In this context, our regression model can be specified as follows:

(1) $$y_{itm}=\alpha +\tau {\mathrm{MMR}}_{it}+\delta \left({\mathrm{MMR}}_{it}\times {\mathrm{Post}}_{t}\right)+\lambda_{t}+\mu_{m}+\gamma X_{imt}+\epsilon_{itm},$$

where $y_{itm}$ represents the short- and long-run outcomes for child *i* born in year *t* to mother *m*. The right-hand side variables include a constant α, an indicator for individual immunization status for ${\mathrm{MMR}}_{it}$ and its interaction with an indicator variable ${\mathrm{Post}}_{t}$ that equals one if the child is born on or after 1987. The regression also controls for birth year fixed effects $\lambda_{t}$, sibling fixed effects $\mu_{m}$, a vector of controls $X_{imt}$ including sibling-varying factors such as birth order fixed effects and gender. We denote the residual fluctuation around the outcome with $\epsilon_{itm}$.

In Equation (1), τ shows the effect of the vaccine for a child born during the high-exposure period, while τ+δ represents the vaccine effect for the no-exposure period. We expect any vaccine effects on child health and later life outcomes to be captured by τ and estimate null effects for the latter. To illustrate the extent of bias in naive estimates, we also present results without sibling fixed effects. Additionally, we provide estimates without $X_{imt}$ to confirm that child-level characteristics do not significantly impact vaccine uptake. Standard errors are clustered at the sibling level to account for within-sibling correlation across our regression outcomes.

The causal interpretation of τ relies on the assumption that the factors driving discordance in MMR immunization status across siblings are not endogenous to health and later-life economic outcomes. In addition, the vaccine should have an impact on our outcomes if the vaccinated individual was born before 1987, with no effect expected for cohorts born after herd immunity was established by the 1986 massive immunization campaign.

## 3. Results

### 3.1. Immunization Campaign and Herd Immunity

**Figure 1:**
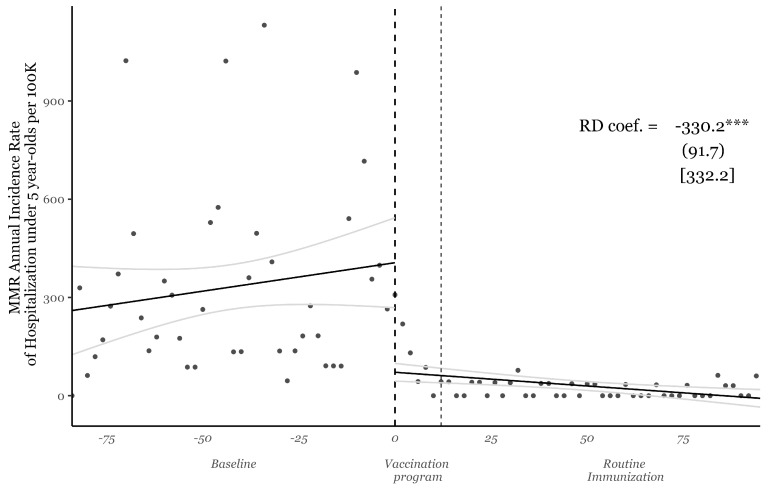
Annualized incidence rate of hospitalization in the under-5 population in Copenhagen and fitted linear trends: 75 months before and after the introduction of the nationwide vaccination campaign (vertical line at 0). The vertical line 12 months after the introduction indicates the transition period during which all children aged 2−12 were offered the vaccine. Regression discontinuity (RD) method estimate the drop in hospitalization incidence rate (see Equation (2)). *** *p* < 0.01.

We begin by analyzing the incidence rates of hospitalizations for the under-5 population in Copenhagen due to measles, mumps, and rubella (MMR) over the 75 months before and 75 months after the initiation of the nationwide vaccination campaign. This descriptive analysis helps us empirically establish the onset of widespread immunity in our sample. Figure 1 shows the monthly time trend of the annualized incidence rate of hospitalization in the under-5 population in our analysis sample from the Copenhagen School Health Records Register (CSHRR), which includes children born in Denmark between 1977 and 1994. The figure is centered around January 1987, the start of the nationwide vaccination campaign. The vertical line around 12 months after the introduction of the vaccine in the DCVP indicates the transition period during which all children aged 2–12 were offered the vaccine.

We also fit linear trend lines for the 75 months before and after the vaccination campaign and provide a regression discontinuity estimate to quantify the sudden drop in hospitalization incidence due to the massive and successful immunization effort. Formally, we estimate the following regression:

(2) $$HR_{t}=\alpha +\beta {\mathrm{Post}}_{t}+f\left(S_{t}\right)+\epsilon_{t}\;\mathrm{for}\;\mathrm{all}\;S_{t}\in \left(c-h,c+h\right),$$

where $HR_{t}$ denotes the incidence rate of hospitalization at time *t*, ${\mathrm{Post}}_{t}$ is a binary variable that equals 1 for the period following the nationwide vaccination campaign and 0 otherwise, and $S_{t}$ represents the running variable calculated as the number of months relative to January 1987. We fit two continuous linear functions, $f(S_{t})$, on either side of the regression sample, encompassing data from *h* months before and after the vaccination campaign. We use an ad hoc bandwidth of 75 months as this specification is primarily intended to distinguish between two distinct periods in terms of widespread herd immunity in Copenhagen. Given the birth cohort structure of the data, we calculated robust standard errors using the Newey–West estimator, which accounts for heteroskedasticity and autocorrelation up to lag 1.

In this model, β captures the abrupt drop in the hospitalization incidence rate attributable to the vaccination campaign in Denmark. Figure 1 clearly illustrates that the immunization campaign was highly successful, nearly eliminating MMR-related hospitalizations. The annualized monthly incidence rate of hospitalization was 332.2 cases per 100,000 children under 5 years old before the campaign, which virtually dropped to zero following the MMR campaign and subsequent routine immunizations, thanks to the establishment of widespread immunity. While these figures pertain to Copenhagen, similar trends and declines are observed in (a population-based version of) nationwide data. Figure 1 using data from Denmark is available upon request from the authors.

Given that measles is highly contagious, achieving and maintaining herd protection is challenging. Although there are still rare and sporadic cases of measles, the data clearly show that measles has been eliminated as a significant contagious disease in Denmark following the vaccination campaign.

These results highlight the two distinct periods that we analyze in our study. Before the vaccination campaign, only those who were individually vaccinated were protected against these viruses. However, after the campaign, the individual vaccination status became less critical due to the immediate establishment of widespread community protection.

### 3.2. Cohort Analysis of MMR-Related Hospitalizations

Next, we focus on our sibling sample to estimate the direct health effects of MMR immunization before and after the vaccination campaign among siblings with discordant vaccination status. The regression results presented in Table 2 illustrate the impact of MMR vaccination on the number of hospitalizations due to measles, mumps, and rubella per 10,000 births within 5 years after birth and between 2 and 12 years of age, across different specifications. Columns (1) and (4) show the baseline OLS estimates, where the model controls only for cohort fixed effects. The coefficient on the vaccine effect for the pre-1987 cohort is significant and negative in both columns, indicating a substantial reduction in hospitalizations during the high-exposure period. Specifically, the estimates suggest that vaccination reduced hospitalizations by 8.65 per 10,000 births within 5 years (column 1) and by 6.66 per 10,000 births within 12 years (column 4).

In columns (2) and (5), we add sibling fixed effects, which are the preferred specifications because they control for unobserved family-level characteristics that may influence both vaccine uptake and health outcomes. The results remain similar, with the vaccine effect for the pre-1987 cohort still significant and slightly larger in magnitude, reducing hospitalizations by 10.64 per 10,000 births for children within the first 5 years of life, and by 8.92 per 10,000 births for children aged 2–12 years.

Columns (3) and (6) further include controls for gender and birth order fixed effects, which show similar results. As in the previous analysis, the relative changes indicate herd immunity for cohorts born after 1987. Compared to the outcome mean, column (3) shows a decrease of approximately 87% (10.61/12.10) within 5 years, and column (6) shows a complete elimination of hospitalization cases within 12 years after birth for cohorts born before 1987.

The coefficients for the post-1987 cohort are not statistically different from zero across all columns, consistent with the expectation that the vaccine’s direct impact would diminish due to the establishment of herd immunity during the no-exposure period. The difference between the pre- and post-1987 effects, captured by the post–pre difference, is significant in all specifications, reinforcing the idea that the vaccine had a substantial protective effect during the high-exposure period but not in the period after the vaccination campaign.

### 3.3. Indirect Effect of MMR Vaccine on Later Life Economic Outcomes

In addition to the documented health benefits of the MMR vaccine, we explore its potential longer-term non-targeted effects on education, labor market outcomes, and income. Table 3 presents the impact of MMR vaccination on educational outcomes for two cohorts: those born before the 1987 vaccination campaign, those born on or after it, and the difference between the two.

For the pre-1987 cohort, the results using sibling fixed effects indicate some improvement in educational outcomes. Specifically, we observe a statistically insignificant reduction of 0.97 to 1.39 percentage points in the likelihood of dropping out before Grade 9; a statistically significant decrease of 2.21 to 2.75 percentage points in the likelihood of not advancing beyond Grade 9 (an 8.2% to 9.8% improvement relative to the outcome mean of 28.06%); and a 2.2 to 2.49 percentage point increase in the likelihood of being active (either employed, in education, or in training), which represents a 2.6% to 2.9% improvement relative to the outcome mean of 84.75%.

For cohorts born after the herd immunity period, there are no significant differences between MMR-vaccinated children and their non-vaccinated siblings. Furthermore, we lack the statistical power to detect a significant difference-in-differences estimate across the outcomes in any of the specifications.

### 3.4. Economic Outcomes Associated with MMR Vaccination

We assess the association of the MMR vaccination with longer-term economic outcomes, including employment, annual wage income, and social benefits, as presented in Table 4. The OLS results without sibling fixed effects again suggest a positive self-selection, particularly for cohorts who were vaccinated when the MMR vaccine was not part of the nationwide immunization program. Estimates for net-of-sibling fixed effects are substantially muted and statistically insignificant for all outcomes and specifications. For older vaccinated cohorts, before 1987, discordant siblings suggest a 1.3 to 1.9 percentage point increase in employment compared to a mean of 49.6%, a 0.5 to 2% increase in real wage income measured at the age of 24, and a 1.6–1.8% decrease in the probability of receiving social benefits at the age of 24. For cohorts born on or after 1987, there is no such consistent pattern across point estimates and outcomes. The difference-in-differences estimates are accordingly unable to differentiate the vaccine effect in two distinct periods in Denmark.

## 4. Discussion

This study investigates the long-term impacts of the MMR vaccine on both health and economic outcomes. By using a unique dataset from the Copenhagen School Health Records Register, we use the variation in the vaccination statuses of siblings between two distinct phases of vaccination in Denmark to account for family-level heterogeneity that could confound the estimated effects of the MMR vaccine.

Our findings underscore the critical role of the MMR vaccine in reducing hospitalizations due to measles, mumps, and rubella among children in Denmark, particularly during the high-exposure period prior to the nationwide immunization campaign in 1987. The introduction of the vaccine not only diminished the immediate health risks associated with these diseases but also contributed to a near elimination of MMR-related hospitalizations post-vaccination, demonstrating the effectiveness of the vaccine in establishing herd immunity.

Beyond the direct health benefits, our study investigates the potential broader socio-economic advantages provided by the MMR vaccine. Our empirical results suggest that MMR vaccination prior to herd immunity was associated with modest improvements in educational outcomes, including a reduction in the likelihood of not completing ten years of education with a final exam before age 16, a decrease in the likelihood of completing only ten years or less of education by the age of 21, and an increase in the likelihood of being employed or pursuing further education, thus avoiding classification as not in education, employment, or training at age 21. We also examine economic outcomes such as employment, real wage income, and reliance on social benefits, which show patterns consistent with the educational findings. However, these estimates lack statistical precision, limiting our ability to draw strong conclusions about the vaccine’s effects on human capital.

Finally, for all human capital outcomes, the association between vaccination and human capital accumulation decreases substantially in magnitude across all outcomes and cohorts when we include sibling fixed effects, which suggests strong positive self-selection by parents of higher socioeconomic status in vaccinating their children.

These results contribute to the growing body of literature examining the effects of the measles vaccine on human capital and labor market outcomes. Using population-level administrative data from Denmark, our analysis offers new insights into the long-term impacts of measles vaccination within the context of a developed country with universal healthcare. Given the limited number of studies on these outcomes and the mixed findings in the existing literature, our investigation provides much-needed evidence to advance the field towards a consensus.

Our investigation differs from the concurrent measles literature in three aspects. First, all previous studies—with the exception ofAnekwe et al. [22]—rely on a research design that leverages variation in pre-vaccination measles infection rates across states or regions and cohort differences in vaccine exposure driven by year of birth in a difference-in-differences framework. In this paper, we use unique data on the actual measles vaccination status of individuals born between 1977 and 1994, which we recently digitized from the Copenhagen School Health Records Register. These data encompass comprehensive health information collected through school examinations. With actual information on the vaccination status linked to rich administrative data obtained from demographic and health registers, our primary strategy for identification is based on a sibling fixed-effects design, in which we compare the long-term outcomes of siblings with discordant vaccination status.

Second, the setting of our analysis differs substantially from those in the concurrent measles literature. Importantly, the measles vaccine was introduced in Denmark in 1987, much later than in most other countries. Unlike the United States, the United Kingdom, and Mexico—where the vaccine was introduced decades earlier in 1963, 1968, and 1973, respectively—Denmark’s later adoption allows for a more contemporary analysis of the effect of the measles vaccine. Therefore, the evidence from the current analysis aligns with more recent changes in both educational systems and labor markets, offering insights that are relevant to the current context.

Third, existing studies exclusively focus on education and labor market outcomes. In contrast, we are equipped with a wide range of health information including hospitalizations from administrative data registers. We use these data to first demonstrate that the sharp declines in hospitalizations due to measles following the introduction of the vaccine as well as to investigate non-specific health effects within a unified framework.

### Limitations

Despite the strengths of our sibling fixed-effects research design—which includes a long period of herd immunity providing a powerful placebo test—this study is not without limitations. One concern is the potential for residual confounding due to unobserved factors that change within families over time. For example, shifts in living conditions, parental preferences, economic fluctuations, differing sibling health conditions (including contraindications to vaccinate), or later social restrictions (e.g., kindergarten or school policies for unvaccinated children) could influence vaccination decisions and potentially bias the results if they are correlated with the outcomes.

For later-life human capital outcomes, such as education, income, and employment, our analysis lacks statistical precision at conventional levels. This is primarily because these outcomes are measured much later in life, making them inherently noisier due to the extended time lag between the cause (vaccination) and the effect. Over this time, various other factors contribute to human capital formation, potentially mitigating any negative effects associated with a lack of vaccination. As a result, isolating the specific impact of vaccination on these outcomes becomes more challenging, leading to less precise estimates. It is important to note that our sample may also be underpowered to capture later downstream outcomes since we only have data until the age of 24 and some individuals may still be enrolled in education, meaning we do not fully capture the long-term effects on economic and labor market outcomes.

Finally, the environment in which children grow up can greatly influence the effects of vaccines. Since our study was conducted in Denmark—a country with a comprehensive healthcare system and low levels of health stressors—it may not fully capture the potential benefits that vaccination programs could have in less-resourced settings. In environments where adequate nutrition is lacking, anemia rates are higher, and other health challenges are more prevalent, the benefits of vaccination may be significantly amplified.

## 5. Conclusions

This study examines the long-term impacts of the MMR vaccine on health, education, and labor market outcomes using a unique dataset and a sibling fixed-effects research design. Our findings confirm the significant role of MMR vaccination in reducing hospitalizations due to measles, mumps, and rubella prior to the establishment of herd immunity. Beyond health, we find that vaccination before herd immunity modestly improved educational outcomes, with positive but statistically imprecise estimates for labor market measures such as employment and labor market income. The evidence also suggests positive self-selection in vaccination, particularly among families with higher socioeconomic status, emphasizing the need to account for such factors when evaluating vaccine effects.

## Figures and Tables

**Table 1:** Descriptive statistics.

	Full Sample		Sibling Sample		Mean Difference
	n	%	n	%	
Child characteristics					
Female	25,906	49.0%	13,958	49.0%	0.0
Birth year, mean (Std. dev.)	1986.1	(5.3)	1986.3	(4.9)	−0.2
Mother characteristics					
Number of children in family, mean (Std. dev.)	1.70	(0.84)	2.36	(0.69)	−0.64
One child	24,347	46.1%	0.00	0.00	46.1
Two children	20,466	38.7%	20,466	71.9%	33.1
Three children	5761	10.9%	5761	20.2%	−9.3
Four children	1624	3.1%	1624	5.7%	−2.6
Five or more children	2242	4.2%	2242	7.8%	−3.6
Married	30,152	57.6%	17,854	63.3%	−5.7
Basic education	22,590	43.1%	12,770	45.3%	−2.1
Vocational or high school	16,618	31.7%	8099	28.7%	3.0
Further education	13,146	25.1%	7334	26.0%	0.9
Age at birth, mean (Std. dev.)	28.32	(20.50)	28.29	(23.16)	0.03
Hospitalizations due to measles, mumps, and rubella (per 100,000 births)					
Ages 0–5, mean (Std. dev.)	6.97	(83.18)	6.24	(78.73)	0.73
Ages 2–12, mean (Std. dev.)	4.37	(65.99)	3.75	(61.12)	0.62
Human capital and labor market outcomes					
Dropping out before grade 9	6320	12.2%	3308	11.8%	0.4
Not advancing beyond grade 9	13,556	26.6%	7334	26.7%	−0.1
Active at age 21 (not NEET)	42,438	83.2%	22,905	83.3%	−0.1
Employed at age 24	22,843	45.4%	12,144	44.7%	0.6
Annual wage income at age 24, mean (Std. dev.)	160,710	(11,877)	159,824	(120,222)	885
Received social benefits at 24	15,541	29.4%	8449	29.7%	−0.3

Notes: Number of observations is 52,816 in the full sample and 27,734 in the sibling sample. NEET is an acronym used to define young people not in education, employment, or training. Annual real wage income is in DKK, adjusted for inflation using 2018 as the base year. Wage information only exists for those with positive values, with 41,945 observations for the full sample and 22,018 for the sibling sample. Comparing the means of the full and sibling samples, the following variables are significantly different at p=0.001: birth year, number of children, mother’s partner status, and education.

**Table 2:** Impact of MMR vaccination on hospitalizations due to measles, mumps, and rubella.

	Hospitalization Within 5 Years				Hospitalization Between Age 2–12		
	(1)	(2)	(3)		(4)	(5)	(6)
Pre-1987 cohort	−8.65 ***	−10.64 ***	−10.61 ***		−6.66 ***	−8.92 ***	−8.93 ***
	[−12.28, −5.02]	[−17.46, −3.83]	[−17.46, −3.76]		[−9.68, −3.63]	[−14.68, −3.16]	[−14.71, −3.15]
Post-1987 cohort	−0.32	1.25	1.23		−0.73	0.33	0.42
	[−1.33, 0.69]	[−2.63, 5.14]	[−2.61, 5.07]		[−1.71, 0.25]	[−2.80, 3.47]	[−2.70, 3.53]
Post–Pre difference	8.34 ***	11.90 ***	11.84 ***		5.93 ***	9.25 ***	9.35 ***
	[4.56, 12.11]	[3.88, 19.92]	[3.82, 19.86]		[2.75, 9.11]	[2.53, 15.98]	[2.62, 16.07]
N	52,816	27,734	27,734		52,816	27,734	27,734
Outcome mean	13.45	12.10	12.10		8.60	7.54	7.54
Clusters (mother)	37,534	12,452	12,452		37,534	12,452	12,452
$R^{2}$	0.01	0.47	0.47		0.01	0.48	0.48
Cohort FE	X	X	X		X	X	X
Sibling FE		X	X			X	X
Gender FE			X				X
Birth Order FE			X				X

Notes: *** *p* < 0.01 [95% CI]. Standard errors are clustered at the mother level. The outcome is the number of hospitalizations due to measles, mumps, and rubella per 10,000 births for children within the first 5 years of life and for children aged 2–12 years.

**Table 3:** The impact of vaccination on the probability of dropping out, not advancing, and being active before and after the introduction of the MMR vaccine.

	Dropping Out				Not Advancing				Active (Not NEET)		
	Before Grade 9				at Age 21				at Age 21		
	(1)	(2)	(3)		(4)	(5)	(6)		(7)	(8)	(9)
Pre-1987 cohort	−2.81 ***	−1.39	−0.97		−7.14 ***	−2.75 **	−2.21 *		4.96 ***	2.49 **	2.20 *
	[−3.69, −1.93]	[−3.28, 0.50]	[−2.86, 0.92]		[−8.46, −5.82]	[−5.19, −0.32]	[−4.64, 0.22]		[3.89, 6.04]	[0.23, 4.75]	[−0.07, 4.46]
Post-1987 cohort	−3.99 ***	−0.52	−0.30		−9.17 ***	−1.55	−1.45		5.74 ***	−0.40	−0.45
	[−5.36, −2.62]	[−3.07, 2.03]	[−2.84, 2.23]		[−10.87, −7.47]	[−4.61, 1.51]	[−4.49, 1.60]		[4.22, 7.27]	[−3.20, 2.40]	[−3.25, 2.35]
Post-Pre difference	−1.18	0.87	0.67		−2.03 *	1.20	0.76		0.78	−2.89	−2.65
	[−2.80, 0.45]	[−2.21, 3.95]	[−2.40, 3.73]		[−4.17, 0.11]	[−2.64, 5.04]	[−3.06, 4.59]		[−1.08, 2.64]	[−6.42, 0.65]	[−6.18, 0.88]
N	51,687	27,108	27,108		50,998	26,465	26,465		50,998	26,465	26,465
Outcome Mean	10.03	9.99	9.99		28.41	28.06	28.06		84.47	84.75	84.75
Clusters (mother)	36,756	12,177	12,177		36,479	11,946	11,946		36,479	11,946	11,946
$R^{2}$	0.01	0.51	0.51		0.01	0.59	0.59		0.01	0.52	0.52
Cohort FE	X	X	X		X	X	X		X	X	X
Sibling FE		X	X			X	X			X	X
Gender FE			X				X				X
Birth order FE			X				X				X

Notes: *** *p* < 0.01, ** *p* < 0.05, * *p* < 0.1. [95% CI]. Standard errors are clustered at the mother level.

**Table 4:** Impact of vaccination on wage income and the probability of being employed and receiving social benefits at age 24 before and after the introduction of the MMR vaccine.

	Employed at 24				Wage Income at 24				Social Benefits		
	(Wage or Self-Employed)				(Annual Earnings > 0)				(Received at Age 24)		
	(1)	(2)	(3)		(4)	(5)	(6)		(7)	(8)	(9)
Pre-1987	1.28 *	1.30	1.88		1839	789	2896		−3.98 ***	−0.46	−0.53
	[−0.15, 2.71]	[−1.56, 4.17]	[−0.97, 4.73]		[−1685, 6023]	[−5954, 10,320]	[−3597, 12,448]		[−5.28, −2.67]	[−3.05, 2.13]	[−3.12, 2.07]
Post-1987	−0.34	0.66	0.81		−3476	−4504	−4226		−7.68 ***	1.07	1.03
	[−2.14, 1.46]	[−2.82, 4.15]	[−2.66, 4.28]		[−6724, 2887]	[−10,913, 8560]	[−10,474, 8932]		[−9.36, −6.00]	[−2.07, 4.20]	[−2.11, 4.17]
Pre-Post diff.	−1.62	−0.64	−1.07		−5314	−5294	−7122		−3.70 ***	1.53	1.56
	[−3.91, 0.67]	[−5.01, 3.74]	[−5.43, 3.29]		[−10,244, 2068]	[−15,786, 9067]	[−17,516, 7122]		[−5.83, −1.58]	[−2.44, 5.50]	[−2.41, 5.53]
N	50,368	25,888	25,888		41,945	19,069	19,069		52,816	27,734	27,734
Outcome Mean	50.6	49.6	49.6		147,545	147,659	147,659		30.3	29.6	29.6
Clusters	36,188	11,708	11,708		31,691	8815	8815		37,534	12,452	12,452
R-squared	0.01	0.55	0.56		0.00	0.57	0.58		0.01	0.55	0.55
Cohort FE	X	X	X		X	X	X		X	X	X
Sibling FE		X	X			X	X			X	X
Gender FE			X				X				X
Birth Order FE			X				X				X

Notes: *** *p* < 0.01, * *p* < 0.1. [95% CI]. Standard errors are clustered at the mother level.

## Data Availability

This paper uses confidential data from Danish administrative registry databases. Therefore, we are not able to make these data publicly available. However, researchers can apply for access to data from Statistics Denmark. Researchers based in institutions outside Denmark can access the data through an affiliation with a Danish authorized research institution. We agree to guiding interested researchers in pursuing these data. In replication files, we will provide all scripts/codes that perform the empirical analyses.

## Abbreviations

The following abbreviations are used in this manuscript:

MMR	Measles, Mumps, and Rubella
CSHRR	Copenhagen School Health Records Register
DVP	Danish Vaccination Program
GP	General Practitioners

## Appendix A

**Table A1:** Number of Discordant Siblings and Discordance Rate.

		Sibling 2		
Pre-campaign		No vaccine	Vaccine	Total
Sibling 1	No vaccine	686	803	1489
	Vaccine	793	3043	3836
	Total	1479	3846	5325
	Discordance rate			0.30
Pre- and post-campaign		No vaccine	Vaccine	Total
Sibling 1	No vaccine	316	719	1032
	Vaccine	777	4404	5181
	Total	1093	5123	6216
	Discordance rate			0.24
Post-campaign		No vaccine	Vaccine	Total
Sibling 1	No vaccine	264	487	751
	Vaccine	471	4411	4882
	Total	735	4898	5633
	Discordance rate			0.17
Full sibling sample		No vaccine	Vaccine	Total
Sibling 1	No vaccine	1142	1756	2898
	Vaccine	1798	10,586	12,384
	Total	2940	12,342	15,282
	Discordance rate			0.23

Note: The table shows the discordance in vaccine status among siblings with the same mother (within mother). The discordance rate is the number of discordant sibling pairs relative to the total number of sibling pairs.
